# Mental Symptoms, Life Satisfaction and Sexual Orientation: A Gender Analysis

**DOI:** 10.3390/jcm12196366

**Published:** 2023-10-05

**Authors:** Roberto Matías, M. Pilar Matud

**Affiliations:** Department of Clinical Psychology, Psychobiology, and Methodology, Universidad de La Laguna, 38200 San Cristobal de La Laguna, Spain; alu0101298450@ull.edu.es

**Keywords:** sexual orientation, gender, life satisfaction, self-esteem, somatic symptoms, depressive symptoms, anxiety, social dysfunction

## Abstract

Research has revealed that homosexual and bisexual people are at higher risk of mental health problems than heterosexual people. However, most studies have focused on differences in disorders and have not examined the intersection of gender and sexual orientation. The main aim of this study is to investigate the relevance of sexual orientation in women’s and men’s mental symptoms, life satisfaction, and self-esteem. A cross-sectional study was conducted with 309 women and men who were homosexual or bisexual and 309 women and men who were heterosexual, aged between 17 and 54. All participants were assessed by four self-reports measuring mental symptoms, life satisfaction, self-esteem, masculine/instrumental and feminine/expressive traits, and traditional gender role attitudes. Results showed an interaction between sexual orientation and gender, with lesbian or bisexual women scoring higher in severe depression symptoms and lower in life satisfaction than heterosexual women. Homosexual and bisexual people scored higher than heterosexuals in somatic symptoms, social dysfunction, and in self-esteem. Women scored higher than men in somatic, anxiety, and insomnia symptoms and in feminine/expressive traits, whereas men scored higher than women in traditional gender role attitudes. We conclude that sexual orientation and gender are relevant to the mental health and well-being of people.

## 1. Introduction

Mental disorders are among the top ten causes of burden worldwide [1], accounting for at least 18% of the global disease burden [2]. The most common mental disorders were depressive disorders and anxiety disorders, and both were more common in women than men [1]. Mental disorders are associated with different genetic, biological, and social factors, and their prevalence and impact are determined by each factor and the interaction between different factors [2]. There is evidence that social determinants affect mental health, and the World Health Organization (WHO) insists on the need to follow a biopsychosocial paradigm in order to face the challenges of mental health [3]. Social determinants of health refer to the conditions in which people live, are born, grow, age, work, and the wider set of forces and systems shaping the conditions of daily life [4,5]. These forces and systems include economic policies and systems, social norms, development agendas, political systems, and social policies [5]. Social risk factors can be at the community level and at the individual level, including a series of attributes such as gender, income, and educational attainment that determine an individual’s position in hierarchies of power, social status, and economic resources [6]. Experiences of discrimination, racism, and historical trauma are also important social determinants of health for certain groups [7].

Gender is an important social determinant of health. According to the American Psychological Association “*Gender* refers to the attitudes, feelings, and behaviors that a given culture associates with a person’s biological sex”, whereas “*Sex* refers to a person’s biological status and is typically categorized as male, female, or intersex (i.e., atypical combinations of features that usually distinguish male from female) [8] (p. 11). Gender refers to the culturally defined roles, behaviors, responsibilities, activities, attributes, and prerogatives associated with being (or being seen as) a woman or a man. It is based on and entails power relations between and among men and women [9,10]. Gender relations of power constitute the fundamental causes of gender inequality and are among the leading social determinants of health [11]. Although, as Heise et al. [10] argue, gender is not captured exactly by the traditional male-female dichotomy of sex but is a complex social system that structures the life experiences of human beings, gender is generally conceptualized exclusively as a trait or identity, with men considered to be (and should be) masculine and women considered to be (and should be) feminine, being masculinity and femininity cultural stereotypes to which people must conform [12]. Femininity is linked to an expressive orientation, with communion being central, defined as focusing on others and their well-being. Masculinity is associated with an instrumental orientation, in which agency is central, characterized by a focus on the self and one’s own mastery and goal attainment, prioritizing independence [12,13,14]. Males have been found to focus on status, power, and achievement through competition more than females [15]. Gender stereotypes present women and men as complementary: men are perceived to be agentic but not communal, whereas women are perceived to be communal but not agentic [16]. Gender norms refer to societies’ spoken and unspoken rules about the acceptable behaviors of girls and boys, women and men, how they should act, how they should look, and even how they should think or feel [17]. Sometimes norms can be so pervasive that individuals mistakenly assume that they are “natural” or “ordained” and therefore immutable [18]. These norms “sustain a hierarchy of power and privilege that typically favors that which is considered male or masculine over what is female or feminine, reinforcing a systemic inequality that undermines the rights of women and girls and restricts opportunities for women, men, and gender minorities to express their authentic selves” [10] (p. 2440). Non-conformity and transgressions of gender norms can trigger negative sanctions [17]. Traditional masculinity entails heterosexuality and the denial of femininity, including negative attitudes toward gay men. It also implies that “real men” should be at the top of the social hierarchy [19]. “Together, these two ideas function to keep a positive male identity by helping men maintain their group distinctiveness and social status” [19] (p. 2).

Most gender systems grant less legitimacy to gender identities or expressions that do not conform to the strict dichotomy of acceptable behaviors for women and men, tending to reject masculinity in women and femininity in men as well as non-conventional gender identities; and individuals who deviate from prevailing gender expectations can undergo discrimination and social sanctioning, which create potent pressures to conform [10]. Restrictive gender norms and gender inequalities are mirrored, reinforced, and perpetuated by health systems, compromising the health and well-being of communities [20]. Gender intersects with other variables, including economic inequality, ethnic or racial hierarchy, caste domination, and differences based on sexual orientation [11]. Gender and social inequalities intersect and multiply their negative effects [20].

According to Darmstadt et al., sexual orientation refers to “an enduring pattern of emotional, romantic, and/or sexual attraction to men, women, or both sexes” [18] (p. 2376). It also refers to “an individual’s sense of personal and social identity based on those desires and attractions, behaviors expressing them, and membership in a community of others who share them” [21] (p. 311). Heterosexuality has been considered “good”, “normal”, and “natural” by society [22], and sexual minorities are viewed as non-normative. Heterosexism is a structural phenomenon that “operates through at least two general processes: First, because everyone is presumed to be heterosexual (a tacit belief often referred to as “The Heterosexual Assumption”), sexual minorities generally remain invisible and unacknowledged by society’s institutions. Second, when sexual minorities become visible, they are problematized; that is, they are assumed to be abnormal, unnatural, requiring explanation, and deserving of discriminatory treatment and hostility.” [23] (p. 19). Research has revealed differences in attitudes towards sexual minorities, with heterosexual men expressing more negative attitudes than heterosexual women. In addition, other variables are also relevant, including adherence to traditional ideologies of family and gender [24,25]. Traditional gender ideologies are prevalent in societies and propose deep and persistent differences between women and men [24]. Many measures of gender ideology have focused on assessing individuals’ level of support for a division of family responsibilities and paid work, which is based on the belief in gendered distinct spheres [26]. These beliefs have been referred to using a variety of phrases, including gender role attitudes, attitudes about gender, gender-related attitudes, and others [26]. Gender differences have been observed, with men holding more traditional gender views than women [27,28].

Social determinants of health also include experiences of discrimination [7]. Despite changes in heterosexuals’ attitudes toward sexual minorities, advances in legal rights, and growing acceptance of same-gender couples in many countries, prejudice against sexual minorities persists [21,23]. Prejudice is manifested in a wide range of behaviors, extending from verbal expressions of dislike to violent assaults [25]. Sexual minority individuals are exposed to repetitive stressors such as social and institutional discrimination, prejudice, and even violent victimization [29,30,31,32]. Research has revealed that homosexual and bisexual people are at higher risk of mental health problems than heterosexual people [33,34,35,36,37], and they are at greater risk of experiencing symptoms of anxiety and depression [30,31]. Minority stress theory explains the excess prevalence of mental health problems among sexual minorities in terms of the stressful social environment produced by stigma, prejudice, and discrimination [29,30,31]. Minority stress includes distal stressors, such as discrimination and victimization, and proximal stressors, such as expectations of rejection, shame, identity concealment, and internalized stigma [29,31,38]. Minority stress can also have a negative impact on self-esteem as a consequence of perceived stigma or discrimination and as a consequence of proximal minority stressors such as internalized homophobia [39]. In a systematic review and meta-analysis, Bridge et al. [39] found that self-esteem tended to be lower in sexual minorities compared with heterosexual individuals.

Even though sexual orientation “is separate from gender identity or how a person chooses to display gender through their appearance, dress, and actions” [18] (p. 2376), descriptive stereotypes of gay men and lesbian women do not conform to the descriptive stereotypes of heterosexual people [40]. Implicit inversion theory assumes that homosexuals are similar to the opposite sex heterosexuals [41], and the literature suggests that gender stereotypes about lesbian women and gay men tend to be the opposite of those about heterosexual people, so heterosexual women are assumed to be highly feminine, lesbian women are believed to be highly masculine, and heterosexual men are assumed to be highly masculine, but gay men are assumed to be highly feminine [42]. The results have shown that people subscribe to the implicit inversion theory, according to which female homosexuals are believed to be similar to male heterosexuals, and male homosexuals are believed to be similar to female heterosexuals [41,42]. Previous research has found that people’s endorsement of the personality traits stereotypically associated with masculinity and femininity is relevant to their mental health and well-being [43,44,45], although sexual orientation has not been taken into account in such research. Therefore, in the present study, we will include an analysis of the person’s endorsement of masculine/instrumental and feminine/expressive traits.

While research has been conducted on sexual orientation and mental health, studies have generally focused on analyzing the presence of mental health problems, but most have not analyzed the impact of sexual orientation on people’s well-being. Furthermore, most research has not analyzed the intersection of gender and sexual orientation. The main aim of the present study is to investigate the relevance of sexual orientation to women’s and men’s mental symptoms, life satisfaction, and self-esteem. A second aim is to examine the relevance of sexual orientation to women’s and men’s endorsement of the personality traits traditionally considered masculine and feminine, as well as traditional gender role attitudes. The third aim is to determine the relevance of age and educational level to heterosexual and homosexual or bisexual women’s and men’s mental symptoms, life satisfaction, self-esteem, endorsement of masculine/instrumental and feminine/expressive traits, and traditional attitudes toward gender roles. We will also analyze the intercorrelations between mental symptoms, life satisfaction, self-esteem, a person’s endorsement of masculine/instrumental and feminine/expressive traits, and traditional attitudes toward gender roles disaggregated by gender and sexual orientation. The last two aims of the study are exploratory, as, to the best of our knowledge, there are no studies that have analyzed it; therefore, no hypotheses will be formulated. Given that previous research has found that homosexual and bisexual people have a higher risk of mental problems and symptoms than heterosexual people [30,31,33,34,35,36,37], that discrimination and prejudice toward sexual minorities persist [21,23], and that their self-esteem tends to be lower compared with heterosexual people [39], we hypothesize the following:

1. Homosexual and bisexual women and men will present more mental symptoms than heterosexual women and men.
2. Homosexual and bisexual women and men will present less life satisfaction than heterosexual women and men.
3. Homosexual and bisexual women and men will present less self-esteem than heterosexual women and men.

## 2. Materials and Methods

### 2.1. Participants and Procedure

This study used a cross-sectional design, and the participants consisted of 618 people from the Spanish general population aged between 17 and 54 years. Half of them (*n* = 309) described their sexual orientation as homosexual or bisexual, and the other half (*n* = 309) as heterosexual. 48.9% (*n* = 302) of the total sample were men, half of whom (*n* = 151) were homosexual or bisexual, and the other half (*n* = 151) were heterosexual. 51.1% (*n* = 316) of the total sample were women, half of whom (*n* = 158) were homosexual or bisexual, and the other half (*n* = 158) were heterosexual. All participants were volunteers and did not receive financial compensation for their participation. Convenience sampling was used. Access to the sample was through educational and workplace centers in different Spanish localities, as well as through the social network of psychology university students who participated in the data collection. Students were trained in administering the tests and received course credit for their participation. After the student contacted each person and asked for their collaboration, if the person agreed to participate, they were given an envelope with the self-administered tests along with instructions for completion and the informed consent form, and both persons met at the same place where the tests had been delivered to pick them up 7 or 10 days later, at the participant’s convenience. To protect the anonymity of the participants, no personally identifiable information was recorded, and the participant was not asked to sign the informed consent form.

Given that the social determinants of health at the individual level include sociodemographic characteristics [6], we controlled for differences in these variables according to gender and sexual orientation. Therefore, we controlled for similar sociodemographic characteristics of homosexual and heterosexual individuals (see Table 1) and for women and men (see Appendix A, Table A1). More than half of the sample were students, and just over a quarter were employed. Most of the participants completed secondary education or professional training, although 21.4% had university degrees. Most of the participants (95.8%) were never married, although almost half had a partner. Only 3.9% of the sample was married or living with their partner.

The sample for the present study was selected from a larger sample of research on gender and health. First, a sample of non-heterosexual people was selected using the following criteria: (1) their sexual orientation was homosexual or bisexual. (2) There were no statistically significant differences between men and women in age, occupation, educational level, or marital status. Once the characteristics of the group of homosexual or bisexual persons were established, the sample of heterosexuals was randomly selected from the large sample using the following criteria: (1) that their sexual orientation was exclusively heterosexual. (2) There were no statistically significant differences in age, educational level, occupation, or marital status between men and women with respect to the homosexual or bisexual group.

The study was conducted in accordance with the Declaration of Helsinki and its later amendments. The questionnaires and scales and a sexual orientation and demographic data collection sheet were self-completed individually and manually, in paper format, with no names or any other data that would allow identification of the participants being recorded. All participants gave their verbal informed consent before completing the questionnaires and scales and were able to cancel their participation in the study at any time. This study forms part of a larger research on gender and health and was positively evaluated by the Ethics Committee on Animal Research and Welfare of the University of La Laguna (study approval number 2013-0058).

### 2.2. Measures

#### 2.2.1. Mental Symptoms

Mental symptoms were measured through the scaled version of the General Health Questionnaire (GHQ-28) [46]. The General Health Questionnaire is a self-administered screening questionnaire and is one of the most commonly used measures of mental health [47]. The 28-item version describes individual health status in terms of four dimensions, which are not independent of one another [46], each subscale consisting of 7 items: somatic symptoms, anxiety and insomnia, social dysfunction, and severe depression. Items were scored according to the Likert-type scale that assigns a weight to each score, from 0 to 3, so higher scores indicate a higher level of symptoms. For the current sample, Cronbach’s alpha coefficient for the 7 items of the somatic symptoms subscale was 0.83; for anxiety, and insomnia it was 0.90; for social dysfunction, it was 0.83; and for severe depression, it was 0.92. 

#### 2.2.2. Life Satisfaction

Life satisfaction was measured through the Satisfaction with Life Scale (SWLS) [48]. It is made up of five items developed to assess a person’s overall judgment of his or her satisfaction with life and is considered to be the cognitive component of subjective well-being. SWLS is recommended as a complement to measures that focus on the assessment of negative states because it assesses the positive side of the individual’s experience and emphasizes the person’s own standards of evaluation [49]. Items were rated on a 7-point Likert-type scale from 1 (*strongly disagree*) to 7 (*strongly agree*), where higher scores indicate a greater level of life satisfaction. For the current sample, the Cronbach’s alpha coefficient was 0.86.

#### 2.2.3. Self-Esteem

The Rosenberg Self-Esteem Scale (RSES) [50] was used to assess self-esteem. The RSES is one of the most widely used self-esteem measures and is composed of ten items developed to assess global self-esteem. The response format is a four-point scale from 0 (*strongly agree*) to 3 (*strongly disagree*), with higher scores indicating higher levels of self-esteem. For the current sample, Cronbach’s α was 0.89.

#### 2.2.4. Masculine/Instrumental and Feminine/Expressive Traits

Masculine/instrumental and feminine/expressive traits were assessed using the reduced version of the Bem Sex Role Inventory (BSRI) [51]. The BSRI was based on the conceptualization of “the conventionally gendered -or “sex-typed individual”- as someone whose self-definition and behavior are thoroughly intertwined with the stereotyped definition of gender appropriateness in his or her culture” [12] (p. 119). It is a self-report inventory that assesses the extent to which a person endorses socially desirable personality traits that are stereotypically associated with men and women as self-descriptive. Items consisting of adjectives or short sentences, 10 of which refer to characteristics and traits traditionally regarded as masculine, such as “independent”, “dominant”, “aggressive”, and “willing to take risk” which make up the masculine/instrumental scale, and 10 characteristics traditionally regarded as feminine, such as “warm”, “gentle”, “tender”, and “sensitive to need of others” which make up the feminine/expressive scale. The response format is a 7-point Likert scale ranging from 1 (*never or almost never true*) to 7 (*always or almost always true*). For the current sample, Cronbach’s alpha for the masculine/instrumental scale was 0.77, and for the feminine/expressive scale, it was 0.88. 

#### 2.2.5. Traditional Gender Role Attitudes

We used the Gender Roles Attitudes Questionnaire (ARG-2) [52] to measure traditional gender role attitudes. The ARG-2 is a self-report measure comprising 22 items that assess the extent to which people hold traditional attitudes toward social roles for women and men. Participants were asked to rate each item on a scale from 1 (*strongly disagree*) to 7 (*strongly agree*), with higher scores indicating more traditional gender role attitudes. For the current sample, Cronbach’s α was 0.88.

#### 2.2.6. Demographic and Sexual Orientation Data Collection Sheet

A sociodemographic data collection sheet was used to collect information on sexual orientation (heterosexual, gay, bisexual, other), gender (women, men, other), and sociodemographic characteristics such as age, educational level, occupation, and marital status.

### 2.3. Statistical Analyses

Internal consistency was computed using Cronbach’s alpha coefficient. Descriptive analyses were carried out to recognize the sociodemographic characteristics of the participants. Comparisons between groups for sociodemographic characteristics were computed using Pearson’s Chi-square tests when the variables were categorical and the Student’s *t* test when they were quantitative. To find out if there were differences between heterosexual and non-heterosexual people and between women and men, 2 − 2 between-subjects analyses of variance (ANOVAs) were performed. Independent variables were sexual orientation (heterosexual and homosexual or bisexual) and gender (women and men), and dependent variables were mental symptoms, life satisfaction, self-esteem, masculine/instrumental and feminine/expressive traits, and traditional gender role attitudes. The bivariate associations between the study variables were calculated using Pearson’s *r* correlation coefficient, except for the educational level, where Spearman’s Rho was used as it is an ordinal variable. For all analyses, *p*-values less than 0.05 were considered statistically significant. Statistical analyses were conducted with SPSS 22.0 (IBM Corporation, Armonk, NY, USA) software.

## 3. Results

### 3.1. Differential Analyses

Table 2 displays the results of two-factor ANOVAs with participants’ sexual orientation (heterosexuals vs. homosexuals or bisexuals) and gender (men vs. women) as between-subject factors and mental symptoms as dependent variables. As can be seen, no statistically significant interaction existed between gender x sexual orientation except when severe depression symptoms were considered the dependent variable. When somatic symptoms were considered as the dependent variable, the main effects of gender and sexual orientation were statistically significant. When anxiety and insomnia symptoms were considered as the dependent variable, only the main effects of gender were statistically significant, and when social dysfunction was considered as the dependent variable, only the main effects of sexual orientation were statistically significant. As can be seen in Table 2, women had more somatic, anxiety, and insomnia symptoms than men, and homosexual or bisexual people had more somatic symptoms and more social dysfunction than heterosexual people, although the effect sizes were very small.

**Figure 1:**
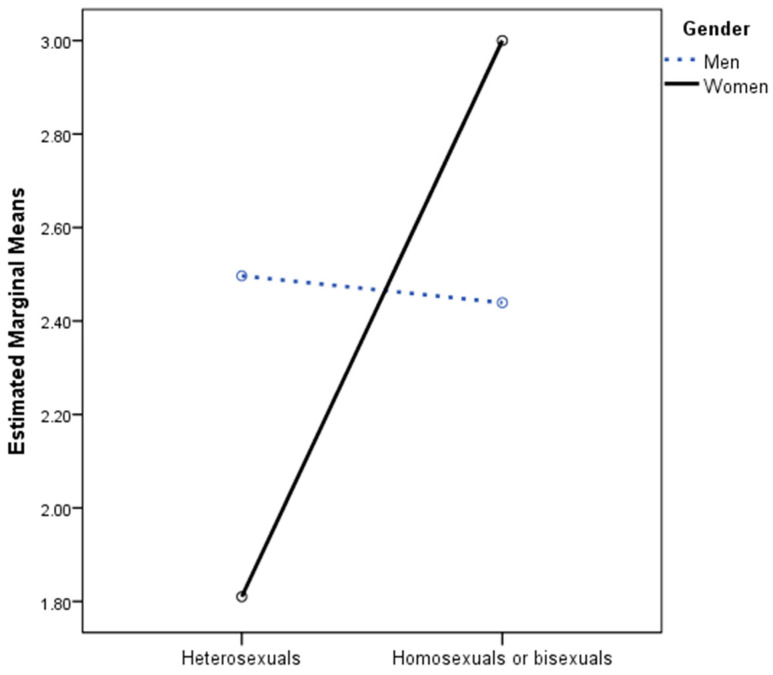
Two-way interaction of sexual orientation and gender predicting severe depression symptoms.

Figure 1 presents the two-way interaction of sexual orientation and gender when severe depression was considered the dependent variable. In the ANOVA conducted in the men’s sample, no statistically significant differences were found as a function of sexual orientation: *F*(1, 297) = 0.02, *p* = 0.899, η_p_^2^ = 0.000. In the women’s sample, statistically significant differences were found: *F*(1, 313) = 7.20, *p* = 0.008, η_p_^2^ = 0.022, although the effect size was small. As shown in Figure 1 and Table 2, lesbian or bisexual women had more severe depressive symptoms than heterosexual women. 

**Figure 2:**
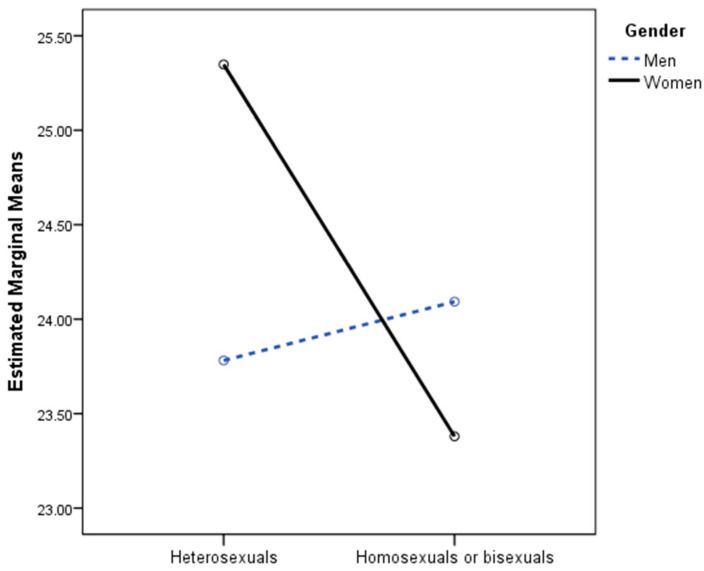
Two-way interaction of sexual orientation and gender predicting life satisfaction.

In the ANOVA in which sexual orientation and gender were considered as factors and life satisfaction as the dependent variable, the gender x sexual orientation interaction was statistically significant: *F*(1, 614) = 6.98, *p* < 0.001, η_p_^2^ = 0.004. In the ANOVA conducted on the men’s sample, no statistically significant differences in life satisfaction were found as a function of sexual orientation: *F*(1, 300) = 0.18, *p* = 0.671, η_p_^2^ = 0.001. In the women’s sample, statistically significant differences were found: *F*(1, 300) = 6.98, *p* = 0.009, η_p_^2^ = 0.022, although the effect size was small. As can be seen in Figure 2 and Table 3, lesbian or bisexual women had less life satisfaction than heterosexual women. 

In the ANOVA in which sexual orientation and gender were considered as factors and self-esteem as the dependent variable, no statistically significant interaction existed between gender and sexual orientation, *F*(1, 612) = 0.01, *p* = 0.97, η_p_^2^= 0.000. The main effect of gender was also not statistically significant: *F*(1, 612) = 1.17, *p* = 0.28, η_p_^2^ = 0.002. Only the main effect of sexual orientation was statistically significant, *F*(1, 612) = 5.93, *p* = 0.01, η_p_^2^ = 0.010, although the effect size was small. As shown in Table 3, homosexuals or bisexuals had lower self-esteem than heterosexuals.

Table 4 displays the results of two-factor ANOVAs with masculine/instrumental and feminine/expressive traits and traditional gender role attitudes as the dependent variables. As can be seen, when a masculine/instrumental trait was considered as the dependent variable, neither effect was statistically significant; neither were the main effects of sexual orientation nor gender, nor was the interaction gender x sexual orientation. When feminine expressive traits and traditional attitudes toward gender roles were considered as the dependent variables, only the gender main effect was statistically significant. As can be seen in Table 4, women scored higher than men in the feminine/expressive trait and lower in traditional gender role attitudes, although the effect sizes were small.

### 3.2. Correlations between Study Variables and with Age and Educational Level

Table 5 presents bivariate correlations of study variables with age and educational level, disaggregated by sexual orientation and gender. As can be seen, although the effect size is small, there are some statistically significant correlations. In heterosexual women, older age was associated with less social dysfunction, more traditional gender role attitudes, and a higher feminine/expressive trait, while a higher educational level was associated with a higher masculine/instrumental trait. In lesbian or bisexual women, older age was associated with fewer anxiety and insomnia symptoms, higher self-esteem, and higher traditional gender role attitudes, whereas a higher educational level was associated with fewer mental symptoms and higher self-esteem. In gay or bisexual men, older age was associated with fewer mental symptoms and higher self-esteem, and a higher educational level was associated with less severe depression symptoms. In heterosexual men, older age was associated with higher self-esteem and a higher educational level with a higher feminine/expressive trait.

Table 6 displays bivariate intercorrelations among study variables disaggregated by sexual orientation for the women’s group and Table 7 for the men’s group. As can be seen, in all groups the mental symptoms showed to be intercorrelated (*p* < 0.001), especially somatic and anxiety symptomatology, whose correlation coefficient was higher than 0.73, except in heterosexual women, where it was 0.56. Self-esteem and life satisfaction were highly correlated in all groups, with *r* coefficients ranging from 0.61 in heterosexual men to 0.72 in homosexual or bisexual women and men, and both variables were associated with fewer mental symptoms. In all study groups, higher scores on masculine/instrumental and feminine/expressive traits were associated with greater self-esteem and life satisfaction, although most of the effect sizes were small or medium. Although the effect size was small, there were some statistically significant correlations between such traits and mental symptoms. In heterosexual men, both traits were associated with less mental symptoms. In gay or bisexual men, higher scores on the feminine/expressive trait were associated with less social dysfunction and less severe depressive symptoms, an association that also appears with the masculine/instrumental trait. In both women’s groups, higher scores on the masculine/instrumental trait were associated with less social dysfunction, although the effect size was larger in heterosexual women. In addition, in this group, higher scores on the feminine/expressive trait were associated with less severe depression symptoms. Traditional gender role attitudes seem to be independent of the other variables, except in the two men’s groups, where a higher score on the feminine/expressive trait was associated with less traditional gender role attitudes.

## 4. Discussion

The main aim of the present study was to investigate the relevance of sexual orientation to women’s and men’s mental symptoms, life satisfaction, and self-esteem. The current research follows an intersectional perspective in which sexual orientation and gender are taken into account. This is important because most research has not analyzed the intersection of gender and sexual orientation, so Klysing asserts that “psychological research conducted on general gender groups may therefore only be applicable to heterosexual individuals, while research on homosexual people in general may be applicable mainly to gay men” [53] (p. 1506).

The first study’s hypothesis predicting that homosexual and bisexual women and men will present more mental symptoms than heterosexual women and men was partially supported. Even though effect sizes were small or might even be trivial on somatic symptoms, compared with heterosexuals, we found that homosexual or bisexual people presented more somatic symptoms and more social dysfunction. Furthermore, lesbian or bisexual women had more severe depression symptoms than heterosexual women, while the severe depression symptom score of gay or bisexual men was very similar to that of heterosexual men. 

Although there were no statistically significant differences in anxiety and insomnia symptoms according to sexual orientation, there were differences according to gender, with more anxiety and insomnia in women than in men, even if the effect size was very small. This finding is consistent with previous research, where women have been found to have more anxiety than men [1]. However, while research has found that women also have more depression than men [1], this was not the case in the current study. The gender effect was also statistically significant for somatic symptoms, with more somatic symptoms in women than in men; the effect size of such differences was larger for gender than for sexual orientation. The gender differences found in the current study on the GHQ-28 scales are consistent with those of a Spanish study published several years ago [54], where women were found to score higher than men on somatic and anxiety symptoms, but there were no differences in severe depression or social dysfunction.

We also found partial support for the second hypothesis, which proposes that homosexual and bisexual women and men would present less life satisfaction than heterosexual women and men. In the present study, sexual orientation was found to interact with gender in predicting life satisfaction. Heterosexual women had more life satisfaction than lesbian or bisexual women, while the life satisfaction score of heterosexual people was very similar to that of gay or bisexual men. These results, together with those found in depressive symptomatology, suggest that the impact of sexual orientation on health and well-being is greater in women than in men and that research on the health and well-being of sexual minorities should also take gender into account.

We found support for hypothesis 3, which predicted that homosexual and bisexual women and men would present less self-esteem than heterosexual women and men. This finding is consistent with Bridge et al.’s [39] Systematic Review and Meta-Analysis and supports their assertion that self-esteem could be a potential target for interventions aimed at reducing disparities in mental health between heterosexual individuals and sexual minorities [39].

The second aim of our study was to examine the relevance of sexual orientation to women’s and men’s endorsement of personality traits traditionally considered masculine and feminine and on traditional gender role attitudes. Results showed that sexual orientation was not relevant to the participants’ endorsement of personality traits stereotypically associated with each gender or to the extent to which women and men held traditional gender role attitudes. These results do not support implicit inversion theory or the assumption that gay men and heterosexual women have similar traits, possessing feminine characteristics and preferences, while lesbian women have similar traits to heterosexual men, possessing masculine preferences and characteristics [40].

Furthermore, we did not find gender differences in participants’ endorsement of masculine/instrumental traits, but we found differences between women’s and men’s endorsement of feminine/expressive traits, with heterosexual and lesbian or bisexual women scoring higher than men in feminine/expressive traits. This last result is consistent with the literature that suggests that women view themselves as having higher expressive/communal characteristics than men [45,55,56]. However, while some studies have found that men view themselves higher than women in instrumental/agency characteristics [45,55,56], this latter difference is less consistent. A recently published study about gendered self-views across 62 countries [55] found that, in all countries, women view themselves more communally than men. Although men view themselves as having agency than women, such a difference was found only in 20 of the countries. 

Findings of the current study showed that women, heterosexuals, lesbians, or bisexuals hold less traditional gender role attitudes than heterosexual and gay or bisexual men. These results converge and extend the evidence of previous research where it has been observed that men hold more traditional gender beliefs than women [27,28]. In the current study, traditional gender role attitudes have been shown to be independent of all study variables except age in women and the feminine/expressive trait in men, although effect sizes were medium to low. Older women had more traditional gender role attitudes than younger women, an association not found in men, and men with more feminine/expressive traits had less traditional gender role attitudes, an association not found in women. This suggests that sexual orientation is less relevant to traditional gender role attitudes than gender, although this is an issue that needs to be explored in future research.

The correlational analyses showed that, although the effect sizes were low, there were some statistically significant correlations between educational level and age with the study variables, although these associations differed according to gender and sexual orientation. In this respect, the association between higher education and fewer mental symptoms in lesbian or bisexual women and between older age and fewer mental symptoms in gay or bisexual men stands out. While older age and a higher educational level were associated with higher self-esteem in lesbian or bisexual women, this was not the case in heterosexual women, whereas in both men’s groups, self-esteem was associated with older age. Furthermore, while older age was associated with higher traditional gender role attitudes in women, especially in heterosexual women, in both men’s groups, age was independent of traditional gender role attitudes. Traditional gender role attitudes seem to be independent of the other study variables, except in the two men’s groups, where less traditional gender role attitudes were associated with higher scores on the feminine/expressive trait. These are interesting results that should be further analyzed in future research.

The current study has several limitations. The first is that it is a cross-sectional study, so no cause-and-effect inferences can be established. Second, all measures were obtained through self-report, so the results may be subject to biases, including social desirability. Third, the sample is not probabilistic, and students and never-married people are overrepresented in the sample. Despite these limitations, this study has contributed to the literature by suggesting that gender plays an important role in differences between sexual minorities and heterosexual people in mental health and well-being. Our results indicate that, although sexual orientation is relevant to people’s mental symptoms and well-being, so is gender, with a greater risk for lesbian and bisexual women. Thus, while homosexual people have a higher risk of social dysfunction, somatic symptoms, and lower self-esteem than heterosexuals, lesbian or bisexual women also have a higher risk of major depression and lower life satisfaction than heterosexual women.

## Figures and Tables

**Table 1:** Demographic characteristics of heterosexuals and homosexuals or bisexual women and men.

	Heterosexuals (*n* = 309)		Homosexuals or Bisexuals (*n* = 309)		χ^2^-Value
	*n*	%	*n*	%	
*Educational level*					
Elementary studies	29	9.4	30	10.4	0.17
High school degree or professional training	212	69.1	196	68.3	
University degree	66	21.5	61	21.3	
Non-data	2		22		
*Occupation*					
Employed	82	26.5	84	27.5	0.09
Unemployed	26	8.5	26	8.5	
Student	201	65.0	195	63.9	
Non-data	0		4		
*Marital status*					
Never married unpartnered	142	46.7	130	44.7	0.29
Never married with partner	149	49.1	149	51.2	
Married/cohabiting	12	3.9	11	3.8	
Separated/divorced	1	0.3	1	0.3	
Non-data	5		18		
	***M***	***SD***	***M***	***SD***	***t*** **-Value**
Age	25.14	7.52	25.50	7.59	−0.59

**Table 2:** Means (*M*), standard deviations (*SD*) and two-way ANOVA statistics for mental symptoms.

Symptoms	Heterosexuals		Homosexuals or Bisexuals		ANOVA		
	*M*	*SD*	*M*	*SD*	Effect	*F Ratio*	η_p_^2^
Somatic							
Men	5.02	4.16	5.61	3.84	Sex. Orient.	4.09 *	0.007
Women	5.82	3.57	6.52	4.24	Gender	7.15 **	0.012
Interaction Gender × Sexual orientation					G × SO	0.03	0.000
Anxiety and insomnia							
Men	5.44	4.82	6.15	4.90	Sex. Orient.	2.42	0.004
Women	6.37	4.67	6.91	5.53	Gender	4.40 *	0.007
Interaction Gender × Sexual orientation					G × SO	0.04	0.000
Social dysfunction							
Men	7.20	3.64	7.59	2.77	Sex. Orient.	5.23 *	0.009
Women	6.91	3.10	7.74	3.51	Gender	0.07	0.000
Interaction Gender × Sexual orientation					G × SO	0.71	0.000
Severe depression							
Men	2.50	4.21	2.44	3.61	Sex. Orient.	3.19	0.005
Women	1.81	3.10	3.00	4.66	Gender	0.04	0.000
Interaction Gender × Sexual orientation					G × SO	3.86 *	0.006

Notes: Sex. Orient = Sexual Orientation. G × SO = Interaction Gender × Sexual orientation. * *p* < 0.05; ** *p* < 0.01.

**Table 3:** Means (*M*) and standard deviations (*SD*) for life satisfaction and self-esteem.

	Heterosexuals		Homosexuals or Bisexuals	
	*M*	*SD*	*M*	*SD*
Life satisfaction				
Men	23.78	6.40	24.09	6.30
Women	25.35	6.09	23.38	7.12
Self-esteem				
Men	22.04	5.49	20.90	5.97
Women	21.52	5.00	20.41	6.37

**Table 4:** Means (*M*), standard deviations (*SD*) and two-way ANOVA statistics for gender traits and traditional gender role attitudes.

Variable	Heterosexuals		Homosexuals or Bisexuals		ANOVA		
	*M*	*SD*	*M*	*SD*	Effect	*F Ratio*	η_p_^2^
Masculine/instrumental trait							
Men	49.52	7.42	48.59	8.44	Sex. Orient.	2.63	0.004
Women	50.34	7.51	49.17	8.66	Gender	1.19	0.002
Interaction Gender × Sexual orientation					G × SO	1.17	0.002
Feminine/expressive trait							
Men	54.83	9.43	55.67	9.47	Sex. Orient.	0.01	0.000
Women	58.66	7.84	57.98	7.94	Gender	19.25 ***	0.031
Interaction Gender × Sexual orientation					G × SO	0.03	0.000
Traditional gender role attitudes							
Men	42.17	15.77	39.68	17.38	Sex. Orient.	1.71	0.003
Women	34.54	13.57	33.90	12.61	Gender	31.23 ***	0.048
Interaction Gender × Sexual orientation					G × SO	0.60	0.001

Notes: Sex. Orient = Sexual Orientation. G × SO = Interaction Gender × Sexual orientation. *** *p* < 0.001.

**Table 5:** Correlations of study variables with age and educational level.

	Heterosexuals				Homosexuals or Bisexuals			
	Women		Men		Women		Men	
	*Age*	*Education* ^a^	*Age*	*Education* ^a^	*Age*	*Education* ^a^	*Age*	*Education* ^a^
Somatic symptoms	−0.06	−0.09	−0.01	−0.01	−0.07	−0.17 *	−0.17 *	−0.17
Anxiety and insomnia symptoms	−0.10	−0.10	−0.04	0.01	−0.17 *	−0.19 *	−0.22 **	−0.13
Social dysfunction	−0.20 *	0.00	0.04	0.01	−0.02	−0.18 *	−0.17 *	−0.08
Severe depression symptoms	−0.14	−0.15	−0.09	−0.04	−0.09	−0.16 *	−0.19 *	−0.17 *
Life satisfaction	0.14	0.06	−0.12	−0.06	0.00	0.03	0.14	0.09
Self-esteem	0.14	0.01	0.17 *	0.09	0.20 *	0.22 *	0.24 **	0.12
Masculine/instrumental trait	0.13	0.19 *	−0.01	0.04	−0.01	0.06	0.15	0.15
Feminine/expressive trait	0.19 *	−0.10	0.12	0.18 *	−0.03	0.03	0.05	0.11
Traditional gender role attitudes	0.30 ***	−0.09	0.09	−0.02	0.18 *	0.04	0.00	0.07

Notes: **^a^** Coefficients calculated with Spearman Rho; * *p* < 0.05; ** *p* < 0.01; *** *p* < 0.001.

**Table 6:** Intercorrelations for study variables for the women disaggregated by sexual orientation.

	2	3	4	5	6	7	8	9
**Heterosexual women**								
1. Somatic symptoms	0.56 ***	0.39 ***	0.47 ***	−0.37 ***	−0.45 ***	0.02	0.04	0.09
2. Anxiety and insomnia symptoms		0.42 ***	0.54 ***	−0.41 ***	−0.43 ***	−0.05	−0.01	0.13
3. Social dysfunction			0.49 ***	−0.50 ***	−0.47 ***	−0.28 ***	−0.15	−0.04
4. Severe depression symptoms				−0.49 ***	−0.63 ***	−0.07	−0.17 *	0.09
5. Life satisfaction					0.63 ***	0.30 ***	0.25 **	−0.12
6. Self-esteem						0.25 **	0.26 **	−0.09
7. Masculine/instrumental trait							0.33 ***	−0.07
8. Feminine/expressive trait								0.13
9. Traditional gender role attitudes								
**Lesbian or bisexual women**								
1. Somatic symptoms	0.77 ***	0.56 ***	0.58 ***	−0.38 ***	−0.47 ***	−0.09	0.04	−0.06
2. Anxiety and insomnia symptoms		0.55 ***	0.59 ***	−0.43 ***	−0.45 ***	−0.05	0.07	0.01
3. Social dysfunction			0.69 ***	−0.49 ***	−0.55 ***	−0.16 *	−0.08	0.07
4. Severe depression symptoms				−0.54 ***	−0.66 ***	−0.16	−0.05	0.06
5. Life satisfaction					0.72 ***	0.23 **	0.28 ***	−0.12
6. Self-esteem						0.24 **	0.17 **	0.03
7. Masculine/instrumental trait							0.32 ***	−0.01
8. Feminine/expressive trait								−0.04
9. Traditional gender role attitudes								

Notes: * *p* < 0.05; ** *p* < 0.01; *** *p* < 0.001.

**Table 7:** Intercorrelations for study variables for the men disaggregated by sexual orientation.

	2	3	4	5	6	7	8	9
**Heterosexual men**								
1. Somatic symptoms	0.75 ***	0.54 ***	0.54 ***	−0.38 ***	−0.36 ***	−0.19 *	−0.20 *	−0.02
2. Anxiety and insomnia symptoms		0.64 ***	0.67 ***	−0.50 ***	−0.51 ***	−0.23 **	−0.22 **	−0.06
3. Social dysfunction			0.61 ***	−0.51 ***	−0.50 ***	−0.23 **	−0.14	−0.08
4. Severe depression symptoms				−0.62 ***	−0.66 ***	−0.22 **	−0.21 **	0.00
5. Life satisfaction					0.61 ***	0.28 ***	0.26 **	0.02
6. Self-esteem						0.39 ***	0.36 ***	−0.07
7. Masculine/instrumental trait							0.34 ***	0.03
8. Feminine/expressive trait								−0.30 ***
9. Traditional gender role attitudes								
**Gay or bisexual men**								
1. Somatic symptoms	0.74 ***	0.51 ***	0.56 ***	−0.51 ***	−0.58 ***	−0.09	−0.09	0.03
2. Anxiety and insomnia symptoms		0.44 ***	0.59 ***	−0.45 ***	−0.52 ***	0.04	−0.03	0.01
3. Social dysfunction			0.54 ***	−0.45 ***	−0.44 ***	−0.16	−0.18 *	0.02
4. Severe depression symptoms				−0.54 ***	−0.65 ***	−0.19 *	−0.19 *	0.09
5. Life satisfaction					0.72 ***	0.38 ***	0.27 **	−0.13
6. Self-esteem						0.45 ***	0.19 **	−0.04
7. Masculine/instrumental trait							0.23 **	0.01
8. Feminine/expressive trait								−0.27 **
9. Traditional gender role attitudes								

Notes: * *p* < 0.05; ** *p* < 0.01; *** *p* < 0.001.

## Data Availability

The data will be provided by the corresponding author upon reasonable request.

## Appendix A

**Table A1:** Demographic characteristics of men and women.

	Men (*n* = 302)		Women (*n* = 316)		χ^2^-Value
	*n*	%	*n*	%	
*Educational level*					
Elementary studies	28	9.8	31	10.0	0.22
High school degree or professional training	199	69.6	209	67.9	
University degree	59	20.6	68	22.1	
Non-data	16		8		
*Occupation*					
Employed	80	26.5	86	27.6	3.47
Unemployed	32	10.6	20	6.4	
Student	190	62.9	206	66.0	
Non-data	0		4		
*Marital status*					
Never married unpartnered	141	49.8	131	42.0	5.49
Never married with partner	133	47.0	165	52.9	
Married/cohabiting	9	3.2	14	4.5	
Separated/divorced	0	0.0	2	0.6	
Non-data	19		4		
	***M***	***SD***	***M***	***SD***	***t*** **-Value**
Age	25.01	6.77	25.61	8.24	−0.99
